# A framework for enhancing spatial and temporal granularity in report-based health surveillance systems

**DOI:** 10.1186/1472-6947-10-1

**Published:** 2010-01-12

**Authors:** Hutchatai Chanlekha, Ai Kawazoe, Nigel Collier

**Affiliations:** 1 National Institute of Informatics, 2-1-2, Hitotsubashi, Chiyoda-ku, Tokyo, Japan; 2 Tsuda College, 2-1-1, Tsuda-machi, Kodaira-shi, Tokyo, Japan; 3 Japan Science and Technology Agency (JST), 2-1-2, Hitotsubashi, Chiyoda-ku, Tokyo, Japan

## Abstract

**Background:**

Current public concern over the spread of infectious diseases has underscored the importance of health surveillance systems for the speedy detection of disease outbreaks. Several international report-based monitoring systems have been developed, including GPHIN, Argus, HealthMap, and BioCaster. A vital feature of these report-based systems is the geo-temporal encoding of outbreak-related textual data. Until now, automated systems have tended to use an ad-hoc strategy for processing geo-temporal information, normally involving the detection of locations that match pre-determined criteria, and the use of document publication dates as a proxy for disease event dates. Although these strategies appear to be effective enough for reporting events at the country and province levels, they may be less effective at discovering geo-temporal information at more detailed levels of granularity. In order to improve the capabilities of current Web-based health surveillance systems, we introduce the design for a novel scheme called spatiotemporal zoning.

**Method:**

The proposed scheme classifies news articles into zones according to the spatiotemporal characteristics of their content. In order to study the reliability of the annotation scheme, we analyzed the inter-annotator agreements on a group of human annotators for over 1000 reported events. Qualitative and quantitative evaluation is made on the results including the kappa and percentage agreement.

**Results:**

The reliability evaluation of our scheme yielded very promising inter-annotator agreement, more than a 0.9 kappa and a 0.9 percentage agreement for event type annotation and temporal attributes annotation, respectively, with a slight degradation for the spatial attribute. However, for events indicating an outbreak situation, the annotators usually had inter-annotator agreements with the lowest granularity location.

**Conclusions:**

We developed and evaluated a novel spatiotemporal zoning annotation scheme. The results of the scheme evaluation indicate that our annotated corpus and the proposed annotation scheme are reliable and could be effectively used for developing an automatic system. Given the current advances in natural language processing techniques, including the availability of language resources and tools, we believe that a reliable automatic spatiotemporal zoning system can be achieved. In the next stage of this work, we plan to develop an automatic zoning system and evaluate its usability within an operational health surveillance system.

## Background

The International Health Regulations (2005) [1], which entered into force on 15 June 2007, have bound 194 countries around the globe to a new legal framework for the coordination of the management of events that may constitute a public health emergency of international concern. The implementation of this framework has underlined the importance of health surveillance technology, both indicator-based, using structured data collected through routine health surveillance, and report-based, using unstructured text sources. Despite the advances in indicator-based public health surveillance [2,3], public health systems in resource-limited jurisdictions are a significant barrier to compliance in many parts of the world [4-6]. Report-based surveillance systems have become another crucial source of epidemic surveillance to fill this gap. Examples of such systems include MedISys [7], GPHIN [8,9], Argus [10], EpiSpider [11], HealthMap [5], and BioCaster [12,13]. These systems generally look for outbreak signals in a variety of electronic sources, including news wires, official reports, and email, which can provide localized and near real-time data on disease outbreaks [4,14,15]. The unstructured texts that are found are then processed using automatic text mining for outbreak-related information, which are organized and presented to the users. Most systems provide map-based visualization by geocoding the alerts to the country scale, with province-, state-, or city-level resolution for the selected countries [5,7,11-13,16].

The geo-temporal encoding of outbreak reports at a more detailed granularity is one of the key requisites for greater utilization of report-based health surveillance systems, but can now only be achieved with accuracy by hand encoding of reports which is time consuming and expensive. For automatic encoding, current systems tend to adopt ad-hoc strategies, generally in the form of detecting the first disease and location pair that matches the predefined criteria or similar heuristics in order to identify the disease-affected location, and use publication dates as the approximate occurrence time of the outbreak events. Although these strategies are effective in reducing both the computational time and false alarming of outbreaks in irrelevant locations, they may lead to the under-reporting of events or issuance of reports at sub-optimal levels of granularity. This results from a characteristic of the news, in which more detailed information concerning the outbreak is often stated later in the story. In the following discussion, we refer to "high granularity" as spatial attributes of events that can be identified at the provincial- or country-levels (or coarser); and "low granularity" as spatial attributes that can be identified at a more detailed resolution, i.e. the city-level or below.

**Figure 1:**
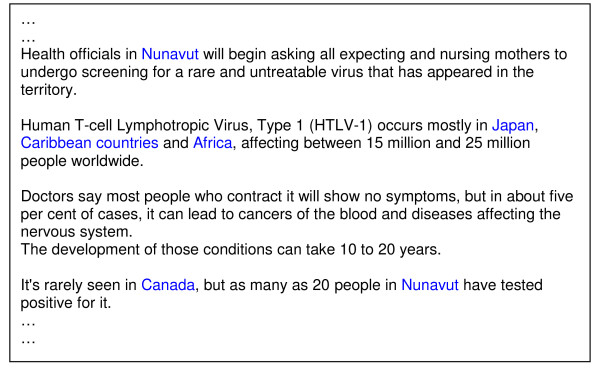
**Various locations with different roles in outbreak news reports**. The example was captured from news article published on CBCnews [51]. The location names occur in the news reports are not always the location of the outbreak. In the text captures illustrated in the figure, *Japan, Caribbean countries*, and *Africa *are referred to as a location where HTLV-1 usually occurs, while *South Africa *and *U.S. *are the countries that provide the medical assistance to the affected country.

In order to improve the performance of current report-based health surveillance systems, we need to go beyond the heuristic methods that analyze only the headlines or the first few sentences of the documents. It has been reported, however, that blindly searching for locations in full text, while increasing the detection sensitivity, can lead to excessive false positives [16]. This is because a news story does not always discuss only the current outbreak-affected location, but can also refer to the locations that are related to the outbreak situation in complex ways, e.g., countries that provide medical assistance, previously affected locations, and so forth. The text capture shown in Figure 1 exemplifies this situation. To effectively identify outbreak locations at lower granularity, a more sophisticated approach that enables systems to distinguish locations where the current outbreak is occurring from other locations must be used. More specifically, the framework must, as a minimum, provide a means to (1) identify outbreak locations at the lowest level of granularity offered by the text, and (2) distinguish newly reported data from historical and hypothetical data.

One existing linguistic-oriented approach that is capable of performing such task is information extraction [17-19], which analyzes documents and extracts outbreak-relevant information, such as the disease, location, and time. However, the inherent problem that any information extraction system generally faces is a trade-off between specificity and sensitivity. Since the low false alarm rate of outbreak detection is very important in health surveillance systems, information extraction used in such systems tends to have a high specificity, which generally leads to a failure in detecting a number of outbreak affected locations. For example, the sensitivity of one reported information extraction system for the outbreak reporting domain was less than 50% [17].

The contribution of this article is to propose a scheme called spatiotemporal zoning, which analyzes each event reported in news articles with regard to its spatial and temporal information, as a means to mitigate the limitations of current report-based surveillance systems by allowing for a fine-grained understanding of the spatiotemporal information of events. Our proposed scheme is represented in the form of a mark-up language that describes the spatial and temporal information of the textual content. Generally, the purpose of mark-up languages is to provide an inter-changeable format for electronic documents, where text content is enclosed by structured text descriptions, called tags. Tags give clear and concise information about the data which they enclose. Within tags, attributes can be given in order to provide additional information about the data. Since the structure of mark-up language must be defined a priori, computer programs can automatically parse marked-up documents and understand the content easily.

In the development of automatic natural language processing systems that involve empirical analysis, annotated corpora have proven themselves to be very important. However, the task of creating large corpora, which generally involves more than one human-annotator, raises concern at least in two respects, which are how to evaluate the annotation scheme and how to assess the reliability of the annotated data. One solution, which has been performed in various computation linguistics tasks, including word sense tagging [20-23], discourse segmentation [24-29], anaphora tagging [30,31] and text summarization [32,33], is to show the inter-annotator agreement. In terms of evaluating the validity of the annotation scheme, the resulting reliability indicates how well the annotation scheme captures the truth of the phenomenon being studied [34]. In terms of assessing data quality, data are considered to be reliable if the annotators can be shown to agree, at a certain level, on the annotation task. The agreement on the annotation results allows us to infer that they share the same understanding, and, consequently, we can expect them to perform consistently under this understanding. The reliability of manually annotated data becomes very important especially when they are used to train a system. If the agreement for the annotation is low, then it is likely that the system may replicate the inconsistent behaviour of human annotators. As the first step of the development of automatic zone annotation, in this article, we focus on the evaluation of the annotated data and scheme based on the inter-annotator agreement. Several metrics are used for measuring the agreement. Higher agreement indicates the more reliable of the annotated data and the scheme.

In this work, we focus on news articles in the English language. However, since our scheme deals with the semantic attributes of events, which are language-independent, we expect it to be readily extensible to other languages.

The remainder of this article is organized as follows. We first provide a concise description of our spatiotemporal zoning and define the events considered within the scope of our scheme. Next, we introduce the spatiotemporal zoning scheme in detail, including the methodology for the scheme evaluation. A quantitative analysis of the evaluation results is then extensively discussed. Finally, we discuss the current limitations of our proposed scheme and the possibility of developing automatic systems based on this scheme. Noted that, most examples used for illustration were drawn from the BioCaster corpus [35].

## Methods

### Task definition

The objective of our spatiotemporal zoning scheme is to enable language technology software to partition text into segments based on the spatiotemporal characteristics of its content. Each segment, which we call a text zone, contains a set of events that occurred at the same geographical location in the same time frame.

**Figure 2:**
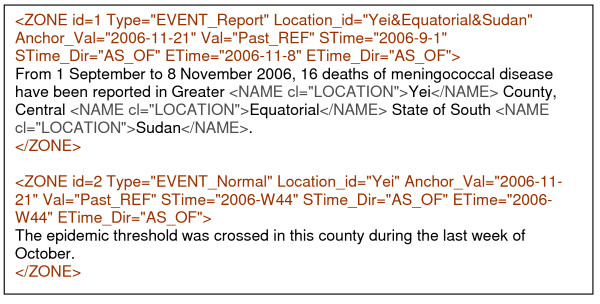
**Text capture of spatiotemporal zoning in a news report**. The example was captured from news published in WHO website. Text is marked-up with spatiotemporal zone according to the annotation guideline. The first zone is report zone consists of one event-predicate, which is "*reported*". This event-predicate event occurred in Yei County, Central Equatorial, in Sudan from 1 September to 8 November 2006. These spatial and temporal information are represented in the zone's Location_ID, STime, and ETime attributes, respectively. The second zone also consists of one event-predicate, which is *crossed*. This event-predicate is annotated as occurred in Yei County, in the last week of October 2006, according to information available in the news report.

The text capture shown in Figure 2 below is an example of our spatiotemporal zoning of the type we envisage in this article.

### Definition of events

Since we are dealing with the analysis of the time and place of events reported in natural language text, it is necessary to explicitly specify the definition of events.

Here, the definition of an event follows the definition used in the TimeML framework [36]. Linguistically, events are considered as predicates describing the states or circumstances in which something changes, obtains, or holds true, and which might need to be located in time. An event is typically defined as a single clause that contains one predicate (i.e. verb) and its arguments (e.g. subject or object).

In our scheme, events may be expressed by

1) Tensed or un-tensed verbs;

2) Certain sets of adjectives, such as "(is) underway" and "(was) ill";

3) Prepositional phrases, such as "(are) on board", "(is) on progress", "(was) in Indonesia".

In the rest of this paper, "event-predicate" means a linguistic constituent consisting of a sentence, finite clause, non-finite clause, or phrase that refers to a single event. Note that, in certain contexts, event-predicate could be interchangeably used to indicate an event that is expressed by the event-predicate. In the following example, expressions marked in bold face represent the event-predicate as described above.

*A 75 year old Canadian ****has contracted ****the virus, most likely when he ****was in New York City ****in early September*.

In the above example, although "was in New York City" can not be qualified as an action in the same way as one might possibly think of "has contracted", but it described the state of the occurrence of the subject, which can change overtime and can be associated with geographical location. So, we regarded it as an event-predicate in our definition.

For the details of the clausal unit qualified for the annotation, please see the Appendix (Additional file 1).

### Basic zone classes

In news report, some text segments convey the contents that cannot be placed in time, i.e. cannot be associated with temporal information. These types of content include sentences that provide general knowledge about certain subjects, or sentences that predict or express the possibility of certain situations. The ability to distinguish event-predicates that express temporally-locatable events from other event-predicates is therefore an essential basic requirement.

In terms of the temporal characteristics, news content can thus be classified into three broad classes, which are described below.

**Generic information:** Text content in this class usually can not be positioned in a specific period along a timeline. There are three major groups of text content that are considered as generic information.

1) General knowledge that is always true or generic events [36]. For example, "*Chikungunya is spread when tiger mosquitoes drink blood from an infected person*."

2) Imperative and interrogative sentences, as well as recommendations, and requests. For example, "*Students with symptoms should stay out of school*."

3) Non-eventive information, which is represented by clauses whose subjects are linked to their predicates (e.g., characteristics, attribute, etc.) via a copula verb. For example, "*The victim is a 12-year-old boy*."

Text content in the second and third groups usually convey information about the current situation, such as the details concerning the victims, control measures, and so forth. In contrast, event-predicates in the first group, i.e., general knowledge, only provide basic information to readers.

**Hypothetical event:** Hypothetical events are those that are alternative or occur in other possible worlds. Event-predicates in this group represent only the perspective or anticipation of the speaker. While Hypothetical events may or may not happen, forthcoming events are those that, without any unexpected circumstances, will definitely occur in the future, such as events that are planned.

**Temporally-locatable event:** Temporally-locatable events are those that have happened, are ongoing, or will definitely happen, and thus, can be located along a timeline. Among event-predicates that represent temporally-locatable events, there is a special subclass of verbs that are usually found in news articles and cause special temporal interpretation of their subordinate event-predicates. These verbs have a communicative function, and we refer to them as 'reporting verbs' [37], such as "say", "tell", "announce", and "report". From a grammatical perspective, the timing of reporting verbs has an influence on the temporal interpretation of event-predicates in the scope of quoted speech. Moreover, there is also the challenge with reporting verbs in deciding whether the time being mentioned is the time of the reporting event-predicate or the time of the event-predicate being reported. Given this characteristic, we believe that it is advantageous to separate reporting event-predicates from other happening event-predicates. For our scheme, we decided to further classify temporally-locatable events into two subclasses: Reporting events and Normal events.

Reporting event-predicates are generally expressed by reporting verbs. Some examples of Reporting event-predicates are shown below:

*(1) The ministry ****said ****the boy might have been infected by sick chickens near his home*.

*(2) "It's very important to test the vaccine on humans and to produce it," Van ****added***.

Normal event-predicates are temporally-locatable events besides Reporting event-predicates. Some examples of Normal event-predicates are;

*(3) A total of 14 of the 19 districts in the state, including Murshidabad*, ***had been affected***.

*(4) Five days after ****returning ****to her hometown of Khon Kaen, she ****fell ill ****with Sars-like symptoms*.

#### ■ Attribute schema

In the spatiotemporal zoning schema, we introduce one attribute for accommodating the event class information.

**TYPE: **This attribute indicates the type of event-predicates in a zone. There are four values for the TYPE attribute. These values are defined according to the classes of the event-predicates. They are: "Event_Info" for the Information class, "Event_Hypothetical" for the Hypothetical class, "Event_Report" for the temporally-locatable Reporting class, and "Event_Normal" for the temporally-locatable Normal class.

As mentioned earlier, events with the Information or Hypothetical type cannot be located along a timeline. As a result, event-predicates with the Event_Info or Event_Hypothetical value for the zone type attribute have no temporal attributes marked in the zone.

### Temporal issue

#### ■ Temporal granularity

In outbreak news, events are usually reported at the level of a 'day' or a coarser period, such as a week, month, or year. In terms of the requirements, organization of the news reports in health surveillance systems with regard to the time is done at the day level, i.e., news is grouped and presented on a daily basis. Given these considerations, in our scheme, temporal attributes are specified at the day level granularity by taking the nearest day to the event occurring time.

#### ■ Attribute design

Events can be either instantaneous or they can occur over a period of time. Thus, representing the occurrence time of events with one attribute may not be sufficiently descriptive. One of the most obvious examples is a report about the repetition or continuation of events over a certain period, as in the following sentence:

*From 1 September to 8 November 2006, 16 deaths of meningococcal disease have been reported in Greater Yei County, Central Equatorial State of South Sudan*.

To enable our scheme to handle these cases, we regard the temporal attribute of the zone as a period with starting and ending times.

Another issue to consider is the relation between events and time. As previously reported [38], events and time can exhibit various relations, e.g., before, after, simultaneous, and so forth, as shown in the example below:

*All patients were admitted to the hospital before 10 January*.

Neglecting the existing temporal relation between an event and the time would result in the loss of detailed information for locating events along a timeline. In order to preserve such information, it is necessary to provide a means to reflect the temporal relation between events and the starting and ending times of the events' occurrence. Two zone attributes can be introduced to express the temporal relation between event-predicates in the zone and the starting time of the occurrence period; and between the event-predicates in the zone and the ending time of the occurrence period.

Another important element is the reference time. Generally, the presence of a reference time is not significant when an event's absolute time can be identified, either from explicitly-stated temporal information or via discourse-level inference. However, we often find cases in which the temporal information is absent or vague as when the occurrence time is represented by means of a verb tense, for example:

*At least 45 people have died of malaria in Jalpaiguri and Coochbehar Districts of North Bengal, senior health department officials said on Thursday*.

In the above sentence, all we know is that the event-predicate "died" started to occur at some time before the utterance time and continued to occur until then, at the very least. In these situations, the reference time plays an important role in the temporal interpretation. Therefore, we include the reference time as one of the temporal attributes in our spatiotemporal zoning scheme.

In news reports, there is no single standard or convention for describing temporal information. The date and time could be referred to as an absolute time, such as "29 Aug 2008", "15/8/2009" or as a relative time, such as "yesterday" or "last Tuesday". These relative forms are less meaningful unless they are interpreted into an absolute time. In order to facilitate further processing and understanding of the event's temporal information, we decided to convert all temporal expressions into a uniform representation. We chose to follow the ISO standard (ISO 8601, the International Standard for the representation of dates and times) for representing time in this work.

#### ■ Attribute schema

According to the issues we have discussed, we defined six temporal attributes for spatiotemporal zone annotation, which are shown below.

**ANCHOR_VAL**: The ANCHOR_VAL attribute is introduced with the purpose of giving a reference time, which is used for interpretation of the other temporal attributes. The ANCHOR_VAL attribute consists of an ISO Normalized form of an anchoring date.

Generally, the default value of ANCHOR_VAL is the document date or news report date. In the case of direct speech constructions, the timing of event-predicates in quoted speech is interpreted with regard to the time of speaking, i.e. the occurring time of the Reporting event-predicate. Therefore, if the event-predicates to be annotated are in the scope of direct speech, the date of that Reporting event-predicate is selected as the value of ANCHOR_VAL.

**VAL**: This attribute was introduced in order to facilitate the systems whose requirements are only to know the approximate occurring time of an event-predicate with regard to the reporting time. The value of the VAL attribute indicates the temporal relation between the reference time, i.e. the value in ANCHOR_VAL, and the time at which the event in focus, which is represented by event-predicate, holds true or happened.

There are three possible values for the VAL attribute: PRESENT_REF for present event-predicate s, PAST_REF for past event-predicate s, and FUTURE_REF for future event-predicate s.

**STIME**: STIME indicates the (approximate) starting time of the event-predicates. The value in STIME is the ISO Normalized form of the temporal information based on the information available in the text. If there is no explicit information indicating the starting time of the event-predicates in the zone, the value in STIME can be: 1) PAST, indicating the event-predicates occurred before the ANCHOR_VAL time, 2) PRESENT, indicating the event-predicates occurred at approximately the same time as the value in ANCHOR_VAL, or 3) FUTURE, indicating the event-predicates occurred after the ANCHOR_VAL time.

**ETIME**: ETIME indicates the approximate ending time of the event-predicate. As with STIME, the value of ETIME can be an absolute or approximate time, e.g. PAST, PRESENT, or FUTURE.

**STIME_DIR**: The STIME_DIR attribute represents the relative direction, i.e. temporal relation, between the value of STIME and the event-predicates in the zone. In the TimeML framework, there are 13 temporal relations between events and temporal expressions or other events [36]. These relations, however, are very detailed. To eliminate unnecessary complexity, we decided to group these relations together under three main classes, which correspond to the possible values of STIME_DIR.

The value of STIME_DIR can be any of the following:

- AS_OF

This class consists of the following types of temporal relations defined in TimeML: "simultaneous", "including", "being included", "during", "being held during", "beginning", "begun by", "ending", and "end by". The AS_OF relation is comparable to the OVERLAP relation in the SemEval-2007 TempEval task [39].

- BEFORE

This class consists of the following types of temporal relations defined in TimeML: "before" and "immediately before".

- AFTER

This class consists of the following types of temporal relations defined in TimeML: "after" and "immediately after".

**ETIME_DIR**: ETIME_DIR is the same as STIME_DIR, except that it represents the temporal relationship between the value of ETIME and the event-predicates in the zone.

### Spatial issue

#### ■ Spatial granularity

The spatial attribute of the event-predicate can be selected from any expression considered to be a location entity according to the BioCaster named entity annotation specification [40]. In the BioCaster project, the location entity is the expression that absolutely refers to the politically or geographically defined location at any granularity. In spatiotemporal zoning, preference is given to the locations with the lowest level of granularity according to the information available in text.

#### ■ Attribute design

It is often that one event-predicate referred to an event that simultaneously occurred in many places. For example, "*Nearly 3,000 tribal people in Ramchandrapur, Ramanujganj, and Wadrafnagar blocks in Surguja district have been in the grip of malaria and typhoid*."

Although multiple locations can be identified to relate to one event-predicate, all of these locations possess the same relation, which is "occur in". Thus, only one zone attribute is required to represent all the locations where the event expressed by an event-predicate occurred.

#### ■ Attribute schema

We define one attribute to represent the spatial information of an event-predicate.

**LOCATION**: The location attribute specifies the geographical location where the events, which are represented by the event-predicates in a zone, happened. The value of the location attribute is the textual form of location as it appears in the documents.

### Zone generation

The task of spatiotemporal zoning can be separated into 3 main steps. (1) Document pre-processing: location names, temporal expressions, and clause boundary in the documents are identified and marked-up. This provides the basic elements for zone attribute analysis and can be done automatically using natural language processing software [41-43]. (2) Attribution annotation: Each event-predicate is analyzed to recognize its class, spatial and temporal attributes. (3) Zone boundary generation: This step is done based on the attribute values of each event-predicate. If the consecutive event-predicates have the same attribute values, they will be merged into a larger zone unit. Otherwise, they will be marked as different zones. To provide further insight into the zone boundary generation task, the process of boundary generation is illustrated in the figure below.

**Figure 3:**
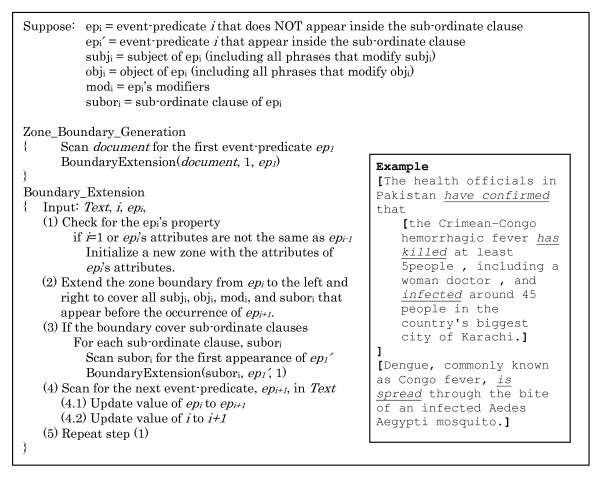
**Zone generation process**. This figure illustrates the algorithm for zone boundary generation. The boundary marked with the square brackets in the text capture is the example of the output from the zone boundary generation process.

As shown in Figure 3 we annotate the text as follows. Start at event-predicate "have confirmed", the boundary of the first zone will be extended to cover the subject (The health officials in Pakistan) and the sub-ordinate clause (the Crimean-Congo ...) of the event-predicate, and then move to the second event-predicate "is spread". Since the class of the second event-predicate is "Information", which is different from the first event-predicate, it is marked up in a new zone. The next step is to analyze the event-predicates inside the sub-ordinate clause. The attributes of "has killed" and "infected" are compatible to each other, so they are marked in the same zone.

Since the zone boundary generation task (3) is relatively trivial when all attributes are known, we focus here on the study and evaluation of attribute annotation (2).

### Scheme evaluation

In the scheme evaluation, we were interested in the reliability of our scheme. This property was evaluated through an inter-annotator agreement, which was done by recruiting a group of annotators to annotate the same set of documents according to the spatiotemporal scheme. After training, three annotators, denoted as A, B, and C, participated in our experiment. The first annotator, annotator A, was the first author of this paper. The second annotator, annotator B, holds a Bachelor of Arts degree. The last annotator, annotator C, was a linguist. The three annotators independently performed a manual annotation on a given document set. In the annotation task, we provided each annotator annotation guidelines and an annotation tool that was developed specifically for this task. This tool is available online [44]. The details of the experimentation data are described below.

### Data collection

The proposed scheme was evaluated on a corpus containing a total of 100 news reports with almost 2000 disease outbreak event-predicates, randomly selected from the BioCaster gold standard corpus [45]. All of the news articles were marked-up with named entity tags and clause boundaries.

**Figure 4:**
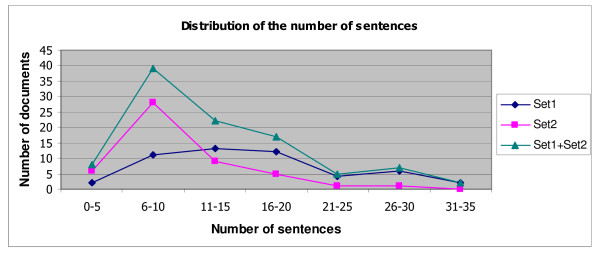
**Distribution of the number of sentences, including partial sentences**. This chart represents the distribution of the number of sentences in our corpus. In corpus set 1, most of the news articles contain 6 to 20 sentences, while in corpus set 2, the highest proportion are the articles that contain 6-10 sentences.

**Figure 5:**
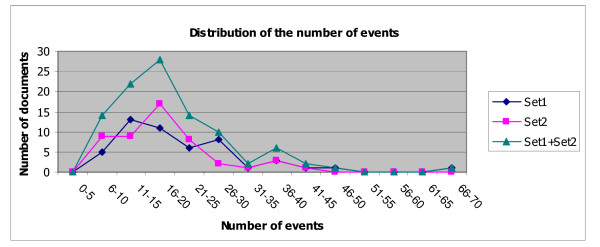
**Distribution of the number of event-predicates to be annotated**. This chart shows the distribution of the number of event-predicates in each document in our corpus. The majority of the documents in overall consist of 6 to 25 event-predicates.

We separated 100 articles into two sets in order to study the inter-annotator agreements between two pairs of annotators. The first 50 files, denoted as Set1, were annotated by annotators A and B. The other 50 files, denoted as Set2, were annotated by annotators A and C. The number of event-predicates and sentences in each document set are shown in Table 1. Figures 4 and 5 show the distributions of the documents with regard to the numbers of sentences and event-predicates that they contain, respectively.

**Table 1:** Data statistics

Corpus	Number of sentences/clauses/phrases	Number of event-predicates
Set1	808	1086

Set2	518	908

**Figure 6:**
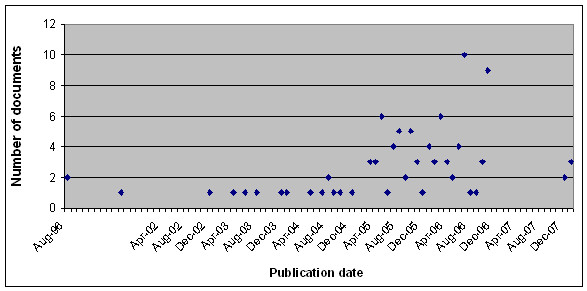
**Distribution of news articles in the corpus by date of publication**. This chart shows the distribution of news articles in our corpus in terms of the publication date. The corpus consists of news articles whose publication dates range from 1996 to 2007. However, the majority of the news articles were published from the middle of 2005 to the end of 2006.

**Figure 7:**
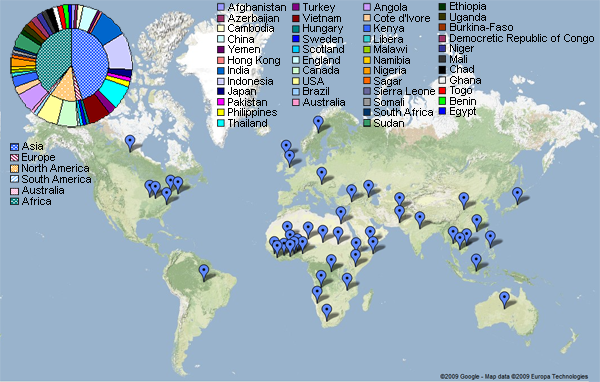
**Distribution of outbreak events reported in our corpus, classified by outbreak-affected country**. This figure represents the outbreak affected countries reported in news articles in our corpus. The map illustration was created by using Google Maps API [52] for the visualized purpose of location distribution. The chart in the top-left corner of the figure shows the number of documents that report the situation in each country. Note that, in our corpus, although most of the articles reported the outbreak within one country, there are also some documents that reported the outbreak situations in many countries.

Figures 6 and 7 represent the distribution of the outbreak news reports in our corpus in terms of the publication date and affected country, respectively. Our corpus covered news articles published from 1996 to 2007, with 44 diseases occurring in 45 countries worldwide. As we would expect, in some articles, one disease outbreak was reported in multiple countries. On the other hand, some articles reported the spreading of multiple diseases within one country.

### Agreement measurement

For quantitative agreement analysis, we used two statistical measures: kappa for evaluating the event class annotation, and the percentage agreement for the spatial and temporal attribute annotation.

#### ■ Kappa

There have been different ways to evaluate the agreement between humans for a task characterized as a mutually exclusive category assignment. Among these, the most widely used are the percentage agreement and Cohen's kappa coefficient [46]. The kappa coefficient, ***K***, is a statistical measure of the inter-annotator agreement for categorical items. It is generally thought to be a better measure of agreement than a simple percentage agreement calculation, since ***K ***takes into account agreements occurring by chance. The equation for ***K ***is;

where Pr(*a*) is the observed agreement among annotators, and Pr(*e*) is the hypothetical probability of a chance agreement. Regardless of the number of annotators, the number of items to be classified, or the distribution of the categories, ***K ***≤ 0 means that there is no agreement other than what would be expected by chance, whereas ***K ***= 1 means that the annotators are in complete agreement.

#### ■ Percentage agreement

In annotating the location and temporal attributes of the marked-up event-predicates, the annotators could freely select an event-predicate's location as any location name appearing in the news report. Since the nature of the task was not exactly a mutually exclusive classification, we decided to use the simple agreement percentage as a measure to show the agreement characteristics between the annotators in assigning the location and temporal attributes. The percentage agreement was calculated by using the below equation.

## Results and Discussion

### Scheme evaluation results

#### Event type annotation

Table 2 lists the proportions of the event-predicates that were classified by each annotator. The trend in the event-type classification was the same for each of the three annotators, for both corpus sets. The number of Normal event-predicates was the highest, followed by the Reporting event-predicates, which we usually found in the context of the reported speech, followed by the event-predicates in the Information and Hypothetical classes.

**Table 2:** Proportions of event-predicates classified by each annotator

Corpus	Annotator	Normal (%)	Reporting (%)	Hypothetical (%)	Information (%)
Set1	A	53.31	23.30	5.80	17.59
	
	B	54.05	24.31	5.80	15.84

Set2	A	50.68	26.75	4.17	18.40
	
	C	49.89	27.53	5.51	17.07

For the event type of annotation, the results showed that our annotation scheme for the zone types is reliable, with ***K ***= 0.87 for annotators A and B, and ***K ***= 0.90 for annotators A and C.

In a mutually exclusive category assignment task, another tool for annotation analysis is the confusion matrix. Table 3 shows the confusion matrices between each of the two pairs of annotators: A and B, and A and C. From the confusion matrices, we can see that the disagreements between annotators A and B and between annotators A and C were found mostly in the classification between the Normal and Information classes (40 times for annotators A and B, and 27 times for annotators A and C). A greater number of disagreements in classifying between the Information and Normal classes could result from the lack of indicative clues. Reporting and Hypothetical classes usually have explicit linguistic signals, such as the presence of certain words, to indicate the class. In contrast, Normal and Information classes do not have such an obvious signal for their classification.

**Table 3:** Confusion matrix between annotators A and B on Set1 and between annotators A and C on Set2

		Annotator A				Total
			
		Normal	Reporting	Hypothetical	Information	
Annotator B	Normal	543	6	11	27	587
	
	Reporting	17	247	0	0	264
	
	Hypothetical	6	0	51	6	63
	
	Hypothetical	6	0	51	6	63
	
	Information	13	0	1	158	172

Total		579	253	63	191	1086

Annotator C	Normal	436	3	2	12	453
	
	Reporting	8	242	0	0	250
	
	Hypothetical	1	0	36	13	50
	
	Information	15	0	2	138	155

Total		460	245	40	163	908

Disagreements between human annotators implicitly indicate hard cases for automatic annotation. To gain insight into the disagreements in the classification of event-predicate, we provide a more detailed qualitative analysis of the disagreements in the event classification task.

1) Disagreements between Normal and Reporting classes

We found that there are certain verbs that usually cause disagreements between annotators. While there is a certain set of verbs that are always considered to indicate Reporting events, such as "say", "inform", and "report", there are also many verbs that can be considered to indicate either Reporting or Normal events, depending on the context. These verbs include "show", "concede", "order", "urge", "recommend", "ask" among others.

2) Disagreements between Normal and Information classes

Disagreements between the Normal and Information classes are the most common among all disagreements. The cause of these disagreements comes mainly from two issues. The first one is the difference in perception of generic and specific events. Event-predicate representing generic events are generally in the form of predicates (i.e. verbs) whose subject argument refers to non-specific entities. However, different annotators might have different views on the predicate's subject in deciding whether it refers to a generic or specific entity. Examples includes; "*People working in the wool industry used to be ****prone ****50 years ago*". In this example, one annotator could consider "*People working in the wool industry*" refers to a specific group of people, while another annotator might consider that it refers to any workers in the wool industry.

The other source of disagreement is caused by the difference in perception between eventive and non-eventive situations. Clauses that describe the attributes or state of entities are considered to indicate the Information class, such as "*The victim is a 12-year-old boy*". We often found, however, that there were many disagreements occurring when clauses are in the form of verb *to be *and a particular adjective, for example; "*A red rash ****is also visible ****on the bodies of the affected persons*."

The above sentence can be paraphrased as "I see a red rash ...". Therefore, this event-predicate could be regarded as representing a Normal event, which expresses a perception of state by the author. We think that this type of sentence is naturally ambiguous as to whether it represents a state or an event.

3) Disagreements between the Normal and Hypothetical classes

Disagreements in this group mainly occurred from confusion between the events that will definitely occur in the future (i.e., expressed by a Normal event-predicate), and a prediction or a conditionally possible event (i.e., expressed by a Hypothetical event-predicate). From error analysis, we found that there were a number of disagreements in deciding whether "would" was used to signal the future aspect or the hypothetical sense, as in the following example:

*The Red Cross said it ****would spend ****nearly one million Swiss francs in a four-month awareness drive*.

4) Disagreements between the Hypothetical and Information classes

Disagreement in terms of the Hypothetical and Information classes occurred very often when there was a hypothetical mention of general concepts or general knowledge, as in the following example:

*Because West Nile virus antibodies can stay within a person's bloodstream for up to 500 days, it ****can be difficult ****to determine the date of infection*.

While one annotator viewed "can be difficult" as indicating Information about the West Nile virus, the other annotator considered it to indicate a hypothetical situation relating to a certain West Nile virus infection.

#### Temporal attribute annotation

Here, we considered an annotation to be temporally-agreed only when all the temporal-related attributes of an event-predicate were consistently marked up by both annotators. The agreement statistics, which were measured by percentage agreement, for temporal attributes are listed in Table 4.

**Table 4:** Agreement statistics for temporal attributes annotation

Annotators	Normal	Reporting	All classes
A and B	0.92	0.97	0.94

A and C	0.95	0.89	0.93

From the results, we can see that the agreement on the temporal attributes was very promising for both pairs of annotators. This indicates that temporal annotation was less confusing for human annotators than location annotation, and that our schemes for temporal annotation were reliable.

In order to locate the cause of disagreement, we once again performed a drill down analysis on the annotated documents. We observed that the disagreements mostly occurred when the temporal information was not directly stated but had to be inferred from the discourse.

**Figure 8:**
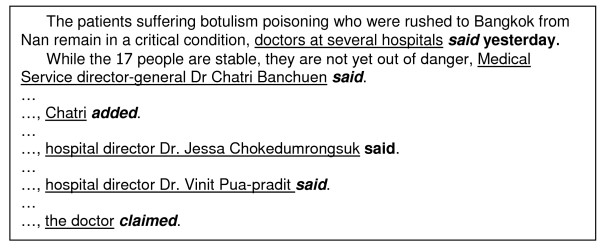
**Example of co-referring of event-predicates**. This example was captured from the news article published on Nation Channel [53]. The captured text shown in the figure exemplifies a situation where multiple event-predicates refer to the same real-world event. In the text example, the phrase "*Medical Service director-general Dr. Chatri Banchuen said*", "*Chatri added*", "*hospital director Dr. Jessa Chokedumrongsuk said*", "*hospital director Dr. Vinit Pua-pradit said*", and "*the doctor claimed*" are parts of the event previously mentioned in the clause "*doctors at several hospitals said yesterday*".

News reports almost always have an abstract at the beginning, which briefly states what happened, together with the location and time of the story's occurrence. In cases where the news reported about an interview with the person in charge, apart from the interview time, the abstract part usually refers to the interviewee by using a short description, such as "senior health officials", instead of their names. This often caused disagreement between the annotators since each annotator might judge differently whether the interviewee appearing later in the story was the same person or was part of a group mentioned in the abstract part. This led to an inconsistency between the annotators in selecting the temporal attributes. Figure 8 shows one example of this situation.

Disagreements were also common when there was a temporal expression in a relative clause, as in the following example:

*(1) It ****had reports ****of 39 deaths from the outbreak of a suspected acute hemorrhagic fever which began in January*.

Here, one annotator felt that the "had reports" event-predicate occurred in the same period as the beginning of the outbreak, i.e. in January, while another annotator thought that the "had reports" event-predicate could have occurred at any time after the beginning of the outbreak.

Differing judgments of the time span or length of an event was another cause for disagreement, as in the example below:

*(2) On Christmas day, a 24-year-old woman from Jakarta also ****died ****from the virus after ****buying ****a live chicken from a market*.

In the above example, while one annotator viewed "buying" as refers to an event that occurred before Christmas day, the other annotator considered both "died" and "buying" to have occurred on the same day, i.e., Christmas day.

#### Spatial attribute annotation

The agreement statistics, represented by the percentage agreement, on the spatial attributes annotation are shown in Table 5, where the agreement values are shown for each event class, as well as the overall agreement.

**Table 5:** Agreement statistics for spatial attribute annotation

Annotators	Normal	Reporting	Hypothetical	Information	All classes
A and B	0.82	0.84	0.80	0.70	0.806

A and C	0.75	0.78	0.58	0.72	0.749

In our scoring method, only the location attributes that were annotated exactly the same by both annotators would be considered to indicate agreement. From the results, we found that the annotators seemed to disagree on the location selection more often for event-predicates in the Hypothetical and Information classes than for event-predicates in the Normal and Reporting classes. For the Information class, disagreements occurred most often when the event-predicate to be annotated consisted of general knowledge, where one annotator considered these event-predicates as world knowledge, and therefore, not specific to any location, while the other annotator considered them as information about specific locations.

In a more detailed analysis, we found that even when the annotators selected different locations, these locations mostly appeared to be related to each other by a partitive relationship. In particular, either the locations selected by one annotator are located within the location(s) selected by the other annotator (such as "Tokyo" and "Japan"), or the locations selected by both annotators are partially the same (such as "Bangkok, Thailand" and "Bangkok"). Although we cannot say that these annotations represent 100% agreement, they are not totally different. As shown in Table 6, with approximate agreement analysis, in which a partial agreement or inclusion of a location is acceptable, the percentage agreement was very high, at almost 100% for most event classes for annotators A and B. The situation was the same for annotators A and C, except for the Hypothetical class, in which the agreement was a little bit lower.

**Table 6:** Agreement statistics for approximate agreement of spatial attribute annotation

Annotators	Normal	Reporting	Hypothetical	Information	All classes
A and B	0.99	1	1	1	0.997

A and C	0.99	0.98	0.89	1	0.986

Although the inter-annotator agreement for exactly-agreed annotation is slightly lower than the inter-annotator agreement of other attributes annotation, in should be noted that the spatial annotations of Normal event-predicates usually had agreement or partial agreement at the state or province level. Especially for the event-predicates that could be regarded as an obvious signal of outbreak situations, such as the event-predicates referring to a spreading of a disease or the deaths of disease victims, the annotators usually had agreement in annotating such event-predicates with the lowest-granularity locations available in the news. This result indicates the promising possibility for identifying outbreak locations with a more detailed geographic resolution, which is a critical area in the future development of effective outbreak detection.

As we examined the raw data to find the characteristics of the disagreements between annotators, we observed that the major source of disagreement came from the spatial information of event-predicates that needed to be recognized via discourse-level inference. Without explicit information at hand, we often found that while one annotator tried to infer the most specific locations according to what was available in the news content, another annotator tended to select locations at a higher level of administration, such as a location at the country or province level, whenever there was uncertainty. The following is an example of these situations:

*(1) Mekong Delta provinces are in the grip of a dengue outbreak with 38% more patients year on year. Measles is also afoot in northern Lai Chau Province. Deputy Minister of Health Trinh Quan Huan announced news of the outbreaks recently, saying that measures ****were underway ****to prevent further spread*.

In the above example, while one annotator selected the Mekong Delta provinces and Lai Chau as the locations of the "were underway" event-predicate, another annotator doubted whether the measures were underway only in these affected provinces, and decided to select Vietnam, which is more general, instead.

There was also a case where a disagreement occurred from the different interpretation of the location of an event-predicate. This kind of situation did not occur very often, but the annotators could sometimes be misled by unclear passages, such as in the following example:

*(2) So far, there's no hint of an outbreak in Canada. But Canadian health officials are watching what happens in the U.S. They may just ****start testing ****birds here to find out if they're carrying the virus. Because if they've ****got it***, *mosquitoes ****will pick it up***, *and then, people ****will be ****next*.

While one annotator considered the event-predicates "start testing", "will pick up", and "will be" related to a hypothetical situation in Canada, another annotator chose the U. S. as the event location.

## Discussion

The investigation brought to light several issues:

■ Event-predicates relating to the spatial movement of an entity (e.g., "transfer", "send", "travel"): Currently, we do not distinguish between the source and destination locations. This information can be critical, however, for detecting international travel health threats. For the next stage of our scheme, we plan to include this information to the scheme.

■ Polarity of event-predicates: This information is necessary in judging whether an outbreak event occurred. However, a sentiment analysis is a very complex task, which is to some extent disjoint to the issues influencing the spatiotemporal semantics [47]. Therefore, in the current scheme, we did not consider the positive or negative sentiments expressed in a sentence.

■ Geographical grounding: Currently, the location attributes are annotated with the surface form of the location names as appearing in the text. In order to effectively analyze and locate events into the geographical references, such as the geographic coordinates, a grounding [48] of these location expressions is necessary. For the next stage, we plan to include this information to the scheme.

Our study on creating a spatiotemporal zoning scheme is a significant step forward towards developing an automatic system using this scheme. The reliability evaluation has provided us with confidence that our annotation scheme and the data produced according to this scheme are reliable and could be effectively used for developing an automatic spatiotemporal zone annotation system. Current advances in natural language processing technologies, previous studies of automatic zoning [49], the promising results for temporal relation identification [39,50], as well as the availability of linguistic tools and resources, can provide a methodology to tackle each sub-problem in spatiotemporal zoning.

## Conclusions

In this article, we proposed a novel zone annotation scheme for partitioning text into segments by means of anchoring event-predicates to their locations and approximate times of occurrence, with the purpose of overcoming the limitation faced in the current report-based health surveillance systems. To evaluate the reliability property of the proposed scheme, we conducted experiments for analyzing the agreements between human annotators. The results of the study are very promising, showing that the proposed scheme is reliable. The inter-annotator scores are more than 0.9 kappa in average for event-type annotation, more than 0.9 percentage agreement for temporal attributes annotation, with a slight degradation in annotating the spatial attribute. In this article, we also addressed the issues that cause disagreements between annotators. This analysis provided us with an insight into the nature of the spatiotemporal annotation task, which assists in the design of automatic annotation methodologies. It is interesting to consider that this might also help to highlight the areas of potential difficulty for human analysts in health surveillance tasks.

We are now developing an automatic zone annotation system capable of annotating news reports according to our proposed scheme and intend to put this into operation in an international media monitoring system. Although we have focused mainly on the analysis of news articles, we believe our approach can be applied to other types of unstructured outbreak-related text, such as official reports and ProMED-mail.

## Competing interests

The authors declare that they have no competing interests.

## Authors' contributions

This work was directed by NC. HC carried out the framework design and analysis with technical support and comments from NC. AK participated in the framework design and provided linguistic support. HC carried out the annotation experiments. All the authors contributed during the whole length of the framework development and in writing this paper. All the authors read and approved the final manuscript.

## Pre-publication history

The pre-publication history for this paper can be accessed here:

http://www.biomedcentral.com/1472-6947/10/1/prepub

## Supplementary Material

Additional file 1 **Appendix: Unit of annotation.** The appendix provides the details of the linguistic units of annotation in spatiotemporal zoning. Click here for file
