# Supplementary material for: A framework for enhancing spatial and temporal granularity in report-based health surveillance systems
Source: BMC Med Inform Decis Mak. 2010 Jan 12;10:1. doi: 10.1186/1472-6947-10-1 (PMC2826291; doi:10.1186/1472-6947-10-1)
Supplement: Additional file 1 — Appendix: Unit of annotation. The appendix provides the details of the linguistic units of annotation in spatiotemporal zoning. [file 1472-6947-10-1-S1.PDF]

Appendix

Unit of annotation

Events can be expressed by various kinds of linguistic component. It is likely that a single sentence may consist of multiple constituents expressing spatiotemporal-separated events. From data observation, we found many cases where a single sentence consists of constituents (e.g. clauses or phrases) pertaining to different event type, or mentioning about events that occurred in different place or time. For example: *The father became ill on 2 July with fever, mild cold, then coughed and was taken to the district hospital on 7 July where he died 10 days after onset.*

In such a case, giving a single annotation to the whole sentence would cause inappropriate aggregation of the spatiotemporal attributes of independent or irrelevant events into a single zone. As a consequence, detailed information, which is possibly important, may be made obscured. The clause-level annotation is considered to be more complicated to the annotator than the sentence-level annotation. However, as a trade-off for the purpose of retaining the detailed information as much as possible, we believe that the clause-level annotation should be more appropriate. Grammatically, a clause is an expression consisting of a subject and a predicate. It is a linguistically stable and easily recognizable unit of annotation for the general readers. Therefore we decide on the clause-level annotation.

As we investigated a number of news reports, however, we found that events conveyed in different types of clauses contribute different levels of importance to the main situation reported in news. For example, noun-modifying clauses (e.g., relative clauses, noun-modifying non-finite clauses, etc.) usually give supplemental information, including possibly unimportant past events. Moreover, these clauses can

be considered as the events that are not in focus from the reporter's perspective. Therefore, we consider it necessary to specify in syntactic terms the constituent types that qualify for an independent zone. With this restriction, we can avoid over-generation of small, scattered zones, which would lead to results that are too complex and unnecessarily detailed. The list of allowed constituent types is shown below. In this list, the square brackets in the examples indicate zone boundaries. Here, A and B are considered as clauses representing events that are either spatiotemporally different or different in type.

1) A sequence of sentences

2) A sentence

3) Coordinating clauses

For example: [A] [and B]

4) Subordinate clause

- Subordinate clause introduced with a subordination marker (e.g., that, whether, and if), for example: [A said that [B].]

- Subordinate clause introduced with a word functioning as the head of the constituent, for example: [A] [when B.]

5) Non-finite clause

- Infinitive, for example: [A [to B].]

If an event expressed by an infinitive cannot be located in time, such as an event expressing a purpose, goal, or intention, we do not consider it to qualify for being in an independent zone.

- Gerund, for example: [A, [saying …].]
